# Proteomic analyses of retina of excitatory amino acid carrier 1 deficient mice

**DOI:** 10.1186/1477-5956-5-13

**Published:** 2007-08-21

**Authors:** Hideaki Okumichi, Takashi Kanamoto, Nazariy Souchelnytskyi, Seiji Tanimoto, Kohichi Tanaka, Yoshiaki Kiuchi

**Affiliations:** 1 Department of Ophthalmology and Visual Science, Graduate School of Biomedical Sciences, Hiroshima University, Japan; 2 Karolinska Biomics Centre, Inst. Oncology Pathology Karolinska University Hospital, Stockholm, Sweden; 3 Laboratory of Molecular Neuroscience, School of Biomedical Science and Medical Research Institute, Tokyo Medical and Dental University, Japan

## Abstract

**Background:**

Excitatory amino acid carrier 1 (EAAC1) is a glutamate transporter found in neuronal tissues and is extensively expressed in the retina. EAAC1 plays a role in a variety of neural functions, but its biological functions in the retina has not been fully determined. The purpose of this study was to identify proteins regulated by EAAC1 in the retina of mice. To accomplish this, we used a proteomics-based approach to identify proteins that are up- or down-regulated in EAAC1-deficient (EAAC1^-/-^) mice.

**Results:**

Proteomic analyses and two-dimensional gel electorphoresis were performed on the retina of EAAC1^-/- ^mice, and the results were compared to that of wild type mice. The protein spots showing significant differences were selected for identification by mass spectrometric analyses. Thirteen proteins were differentially expressed; nine proteins were up-regulated and five proteins were down-regulated in EAAC1-/- retina. Functional clustering showed that identified proteins are involved in various cellular process, e.g. cell cycle, cell death, transport and metabolism.

**Conclusion:**

We identified thirteen proteins whose expression is changed in EAAC-/- mice retinas. These proteins are known to regulate cell proliferation, death, transport, metabolism, cell organization and extracellular matrix.

## Background

Glutamate is an excitatory neurotransmitter but high extracellular concentrations of glutamate leads to neuronal death by apoptosis [1]. Therefore, the glutamate concentration outside neuronal cells must be maintained at low levels, and the concentration is controlled by excitatory amino acid transporters (EAATs). There are five types of EAATs, EAAT1 through EAAT5 [2-5], and the sites of expression of each is different in the central nervous system. Glutamate-aspartate transporter (GLAST) or EAAT1 and glutamate transporter 1 (GLT-1) or EAAT2 are located in glial cells, and GLT-1 is also found in presynaptic terminals. Excitatory amino acid carrier 1 (EAAC1) or EAAT3 and EAAT4 are expressed in the postsynaptic terminals.

In the cat retina, GLAST and EAAT4 are mainly expressed in Mueller cells and astrocytes, and they play a significant role in transporting glutamate [6]. GLT-1 is expressed in bipolar cells, amacrine cells, and retinal ganglion cells (RGCs). EAAT5 is expressed in the terminals of photoreceptor cells. EAAC1 is found in horizontal cells, amacrine cells, and RGCs [6], and it is expressed in Mueller, amacrine, photoreceptor, and bipolar cells in humans and rats [7]. Based on the wide range of expression sites, it is not unreasonable to assume that EAAC1 has many functions in the retina [7]. In addition, the expression of EAAC1 in rat retinal cells in culture increases with high glutamate loading and decreases with low oxygen loading. These findings indicate that EAAC1 is involved in the glutamate transport in the retina [8].

EAAC1 is involved in the neuronal uptake of cysteine which is a substrate for the synthesis of gluthatione, a major anti-oxidant [9]. The activity of EAAC1 is mediated by Akt, a key survival signaling protein [10], and the activity of EAAC1 is regulated by protein kinase C and phosphatidylinositol-3-kinase (PI3K) [11]. Furthermore, EAAC1 has anti-apoptotic activity by controlling XIAP and inhibiting caspase-3 activation [12]. Thus, EAAC1 is not only a glutamate transporter, but also cross-talks with components of intracellular signaling pathways. So, comprehensive analyses are needed to clarify the multiple functions of EAAC1. Here, we report description of thirteen proteins which changes their expression in retina of EAAC1-/- mice.

## Results

### Two-dimensional proteomic maps of EAAC1^-/- ^in mice retina

**Figure 1:**
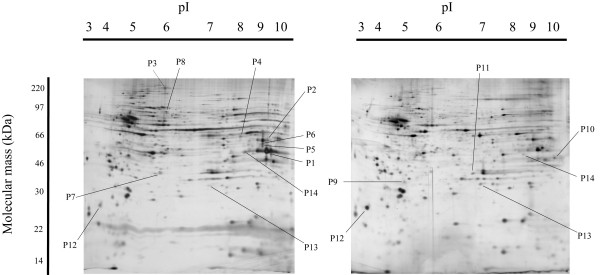
Photographs of two-dimensional electrophoresis gels with annotation of the spots of identified proteins. The left image shows a silver-stained gel of EAAC1^-/- ^mouse retina and the right image is that of a wild-type mouse retina. The proteins spots that increased or decreased in response to the genomic EAAC1, and that were identified by PMF are shown. Spots P1 through P14 represent the annotated spots. The pI gradient of the first dimension electrophoresis is shown on the top of the gels, and the migration of molecular mass markers for SDS-PAGE in the second dimension is shown on the side of the gel. Representative gel images are shown.

To identify the EAAC1-dependent proteins in the retina, we compared the proteome of EAAC1^-/- ^mice retinas with that of wild type mice. Total lysates of each type of retina were resolved by two-dimensional gel electrophoresis. We detected an average of 300 protein spots on the two-dimensional gels after silver staining (Figure 1). We analyzed five gels for each experimental condition to ensure the reliability of the selection of spots with significant changes.

A mother gel was constructed from five gels of EAAC1^-/- ^and ICR mice by PD-Quest software. The volume of all of the protein spots was quantitatively analyzed, and the maximum volume of one spot was 22,000. Weak spots, whose volume in the matched pair spots was less than 2000, were deleted. Next, insignificant spots whose volume difference was less than seven times were also deleted. Thirty-six spots remained, and the gels were directly examined to check that the spots were present in several of the original gels.

Finally, fourteen spots that had increased or decreased volume in EAAC1^-/- ^mice retinas were selected as significant spots, and MALDI TOF mass spectrometry was performed to identify the proteins. [see Additional file 1]

### Clustering of identified proteins

We found that the expression of 9 of the 14 (64%) proteins, P1 through P8 and P14 was increased in the EAAC1^-/- ^mice retina and five (36%) were decreased (Table 1). The expressions of eight proteins, P1 through P8, were undetectable in the ICR control mice and were defined as zero in the PD-Quest-based analyses (+/- in Table 1). On the other hand, three proteins, P9 through P11, were expressed only in the ICR control mice (-/+ in Table 1). Another three proteins, P12 through P14, were expressed in both the EAAC1^-/- ^and the ICR control mice retina. Two spots, P12 and P13, were expressed predominantly in the ICR mice retina, and P14 was expressed predominantly in the EAAC1^-/- ^mice retina.

**Table 1:** Differentially expressed proteins identified by proteomics from retinas of EAAC1^-/- ^(EAAC1KO) and wild type ICR mice retina. a) P1–P14, ID number of spots. b) Probability, sequence coverage, and theoretical value of pI and Mr were obtained from the Pro Found search. The calculation of experimental pI and Mr was based on the migration of the protein on a 2D gel. c) Changes present the EAAC1-/- mice retinal protein volume ratio compared with that of ICR mice. (+/-; EAAC1-/- mice only, -/+; wild type ICR mice only)

a)			b)								c)	
							Theoretical value		Experimental value			Changes
										
	Spot	Protein		Probability	Sequence coverage(%)	ncbi ID	pI	Mr (kDa)	pI	Mr (kDa)		EAAC1KO/ICR
Spots identified only in the EAAC1 KO mice retina												
	P1	gamma-aminobutyric acid (GABA-A) receptor, subunit beta1		1.0e + 000	17	NP_032095	9.1	54.37	8.9	56		+/-
	P2	p300 transcriptional cofactor JMY		1.0e + 000	12	NP_001004185	6.5	82.04	9.2	72		+/-
	P3	LEK1		1.0e + 000	6	AAF07196	4.9	284.46	5.9	210		+/-
	P4	unnamed protein product		9.9e - 001	16	BAB22659	6.0	71.53	7.9	78		+/-
		ANK repeat and LEM domain containing				Q6P1H6						
	P5	unnamed protein product		1.0e + 000	12	BAB30425	10.1	57.84	8.8	66		+/-
		Zin finger 329				NP_080322						
	P6	RIKEN cDNA 2600017A12		1.0e + 000	19	AAH37711	4.3	86.68	8.8	74		+/-
		HIV TAT Spec factor 1				NP_082518						
	P7	striated muscle activator of Rho-dependent signaling		1.0e + 000	34	AAM28877	6.8	42.98	5.9	44		+/-
	P8	Alpha platelet-derived growth factor receptor precursor		1.0e + 000	13	P26618	5.0	123.68	6.0	112		+/-
Spots identified only in the ICR mice retina												
	P9	Galnt7 protein		1.0e + 000	24	AAH49907	6.4	42.70	4.9	34		-/+
	P10	TPA : regulator of sex-limitation 2		1.0e + 000	29	DAA01848	9.8	59.05	9.6	56		-/+
	P11	mKIAA0626 protein		1.0e + 000	21	BAC65614	4.8	39.57	7.1	38		-/+
		microfibrillar associated protein 3 like				NP_082032						
Spots identified in both EAAC1 KO and ICR mice retina												
Spots with high expression in ICR mice retina												
	P12	hypothetical protein		9.9e - 001	23	XP_357614	8.6	27.86	4.0	26		0.11
	P13	unnamed protein product		1.0e + 000	40	BAC26443	12.2	29.52	7.4	29		0.14
Spots with high expression in EAAC1 KO mice retina												
	P14	TPA : regulator of sex-limitation 2		8.8e - 001	23	DAA01848	9.8	59.05	8.3	56		7.06

**Figure 2:**
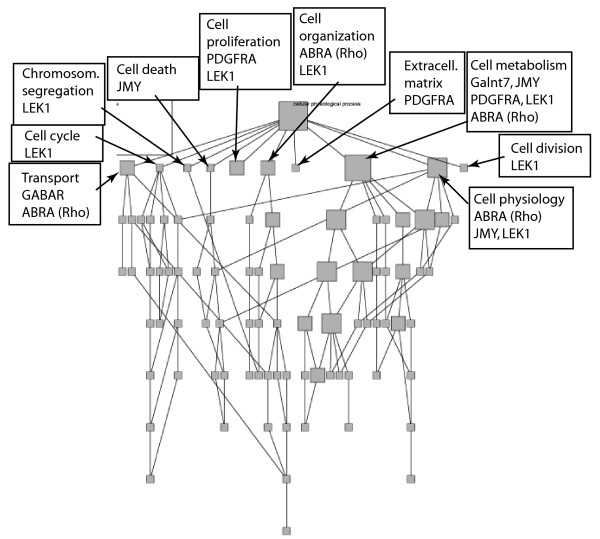
Involvement of the EAAC1-affected proteins in various cellular physiological processes is shown by direct acyclic graph (DAG). Proteins in various nodes are annotated and arrows show corresponding nodes. The first level of affected process is annotated.

Analyses of the identified proteins showed that the absence of EAAC1 has the potential of altering different cellular functions (Table 1). Clustering of identified proteins using GoMiner indicated that ablation of EAAC1 may affect regulation of cell cycle, cell death, cellular organization, metabolism, transport and extracellular matrix (Figure 2). Thus, two cell membrane receptors, five transcriptional factors, one regulator of cell signaling, and one matrix protein were expressed in the EAAC1^-/- ^but not in the wild type mouse retina. PDGFR (P8) and GABAR (P1) have already been identified as signaling proteins and related to EAAC1 function. These findings demonstrated that our approach not only detect previously suggested functional links of EAAC1, but also confirmed the validity of our technique. The identification of TPA, a regulator of sex-limitation 2 in two independent protein spots, P10 and P14, suggests that these proteins are differentially modified post-translationally. The identification of novel proteins open for the possibility of novel mechanism dependent on EAAC1 expression.

## Discussion

The identification of proteins regulated by EAAC1 is important for the understanding of the different functions of EAAC1. We used the proteomic approach and detected thirteen proteins that changed their expression in the EAAC1^-/- ^mouse retina and not in the wild type retina.

There have been reports of a correlation between the PDGFR signaling system and EAAC1. PDGF-BB has been reported to activate EAAC1 through PI3K in C6 gliomas [13], but in EAAC1^-/- ^mice retina, the expression of PDGFR-alpha was altered, which is not a receptor specific to PDGF-BB. It has also been reported that PDGF-trafficking is affected by the C-terminal of EAAC1 [14,15]. Our results showed that the lack of EAAC1 led to an enhanced expression of PDGFR-alpha (Table 1).

GABA is one of the metabolic substrates forming the metabolic cycle of glutamate that is taken in by cells [16]. Thus, the EAAC1-glutamate system is related to GABA, which functions as another neurotransmitter [17]. However, there is a report that EAAC1 is not related to the synthesis of GABA in rat retinas, so it was proposed that EAAC1 and GABAR may form separate signaling responses in the retina from that of the glutamate metabolic system [18].

Proteins related to LEK1, p300-associated protein, proteins related to Rho-GTPase, and Zinc finger protein 329, have been identified as signaling factors (Table 1). LEK1 is a SNAP-25 binding protein which is involved in vesicle-recycling within the cells [19], and it also plays an important role in cardiogenesis and development [20], but its function in the nervous system is still undetermined. p300 is an important transcription factor in several signaling systems [21], and the expression of the co-activator of p300 is increased in EAAC1^-/- ^mouse retina.

Expression of the activator of Rho-dependent signaling, which is a part of the Rho-GTPase family and controls the cytoskeleton organization [22], was also increased in EAAC1^-/- ^mice retina. Zinc finger protein 329 has an effect on neuronal differentiation [23] but its relationship to EAAC1 is unknown.

Several proteins with not explored functions and no previously reported connections to EAAC1, e.g. Galnt7 [24], microfibrillar associated protein 3, ANK repeat, and LEM domain-containing protein.

## Conclusion

Our proteomic study has identified thirteen proteins which change their expression in EAAC1-/- mice retina, as compared to normal retina. Functional clustering of these proteins indicated that EAAC1 may affect various cellular functions.

## Methods

### Animals

EAAC1-deficient (EAAC1^-/-^) mice (on ICR mice background) [25] were kindly provided by Dr. Kohichi Tanaka (Tokyo Medical and Dental University). For controls, wild type ICR mice were purchased from CLEA Japan, Inc (Tokyo, Japan). All animals were maintained in clear plastic cages with standard 12:12 light:dark cycle and handled in accordance with the Guide for the Care and Use of Laboratory Animals by the USA National Institutes of Health.

### Sample preparation

Mice were anesthetized with diethylether, and the eyes were enucleated. The retinas were carefully isolated from the choroid in phosphate-buffered saline (PBS). The isolated retinas were solubilized in sample buffer [8 M urea, 4% CHAPS, 0.5% dithiothreitol (DTT), IPG buffer, pH 3–10], and aliquots of the lysates were stored at -70 degrees. The protein concentration was measured by the Bradford assay.

### Two-dimensional electrophoresis

Two-dimensional electrophoresis and protein identification were performed as described in detail [26]. Isoelectrofocusing was performed on the strips with an immobilized pH gradient (pH 3–10 non-linear gradient, 18 cm: GE Healthcare). First-dimension isoelectrophoresis was performed in IPGphor (GE Healthcare) according to manufacturer's instructions. After the isoelctrofocusing, the strips were placed in equilibration buffer-1 (50 mM Tris-HCl, pH 8.8, 6.0 M urea, 2.0% SDS, 30% glycerol, 1% DTT) and then in equilibration buffer-2 (50 mM Tris-HCl, pH 8.8, 6.0 M urea, 2.0% SDS, 30% glycerol, 4% iodoacetamide). The equilibrated strips were loaded onto SDS-containing 12% polyacrylamide, and SDS-polyacrylamide gel electrophoresis (PAGE) was performed.

After the electrophoresis, the gels were fixed in 7.5% acetic acid and 20% methanol, and sensitized in 25% ethanol, 0.2% sodium thiosulfate, and 3.4% sosium acetate. The gels were then stained with 0.25% silver nitrate and developed with 2.5% sodium carbonate and 0.04% formaldehyde.

### Gel analyses

Silver-stained gels were scanned by an image scanner (EPSON) and analyzed with calculation of the volumes of the spots with the PD-Quest software (BioRad) following the manufacturer's instructions. Five gels from each type of mouse were prepared and a master gel was generated for each type of mouse. The values of the volume of each matched spot on the master gels were compared. Spots with differences in expression were then identified by mass spectrometry.

### Protein identification

The excited protein-containing spots were destained with 30 mM potassium ferricyanide and 100 mM sodium thiosulfate. Then, the gel pieces were dipped in 0.1 M sodium hydrocarbonate and washed with acetnitril. After the gel pieces were dryed, in-gel digestion was performed with trypsin. Then, 10% trifluoroacetic acid (TFA) and acetonitrile were used to extract the peptides, and the extract was desalted on a nano-column. After washing the column with 0.1% TFA, the matrix was eluted with acetnitril containing alpha-cyano-4-hydroxycinnamic acid directly onto the MALDI target. Spectra were generated on a MALDI-TOF-MS (Bruker Daltonics). The spectra were internally calibrated using known internal tryptic peptides from trypsin and searches were made in the NCBI sequences using ProFound. The search results were evaluated by considering the probability, the Z-value, peptide coverage, and correspondence to experimental pI and molecular mass.

### Functional clustering

For functional clustering, proteins were annotated in Gene Ontology [27] and subjected to a search using GoMiner tool [28].

## Competing interests

The author(s) declare that they have no competing interests.

## Authors' contributions

OH carried out the proteomic studies. TK carried out the proteomic studies, data analysis, and participated in drafting the manuscript. NS carried out data analysis. ST and KT participated in the sample reparations. YK participated in drafting the manuscript. All authors approved the final manuscript.

## Supplementary Material

Additional file 1 Protein volume of significant spots. The data provide the actual protein volume of significant protein spots, measured by PD-Quest software. Click here for file
