# Supplementary material for: Proteomic analyses of retina of excitatory amino acid carrier 1 deficient mice
Source: Proteome Sci. 2007 Aug 21;5:13. doi: 10.1186/1477-5956-5-13 (PMC2014740; doi:10.1186/1477-5956-5-13)
Supplement: Additional file 1 — Protein volume of significant spots. The data provide the actual protein volume of significant protein spots, measured by PD-Quest software. [file 1477-5956-5-13-S1.pdf]

	Experimental volume	
Spot	EAAC1KO	ICR
P1	5111	0
P2	3698	0
P3	3615	0
P4	2972	0
P5	2250	0
P6	2174	0
P7	2137	0
P8	2059	0
P9	0	2261
P10	0	3025
P11	0	4155
P12	2053	18627
P13	571	4110
P14	6350	899
